# Supplementary material for: Optimizing expression of Nanobody® molecules in Pichia pastoris through co-expression of auxiliary proteins under methanol and methanol-free conditions
Source: Microb Cell Fact. 2023 Jul 22;22:135. doi: 10.1186/s12934-023-02132-z (PMC10362571; doi:10.1186/s12934-023-02132-z)
Supplement: Supplementary file 1 — Additional file 1: SDS-PAGE analysis of 5 Nanobody molecules (D, E, F, G and H) used to benchmark the methanol-free platform. The Nanobody formats were expressed in the methanol-based system co-expressing RPP0, KAR2, PDI1 and HAC1 (“NRRL Y-11430+RPP0+KAR2+PDI+HAC1”) and compared with the methanol-free system using the wild-type P. pastoris (“NRRL Y-11430 MeOH-free”) and the methanol-free platform strain co-expressing HAC1 (“NRRL Y-11430+HAC1 MeOH-free”). An asterisk (*) marks the band corresponding to each Nanobody format. Indicated titers as measured in the cell broth by PA-HPLC are shown at the bottom of the figure. [file 12934_2023_2132_MOESM1_ESM.docx]

Additional file 1.

|  | **Nanobody D** | | | **Nanobody E** | | | **Nanobody F** | | | **Nanobody G** | | | **Nanobody H** | | |  |
| --- | --- | --- | --- | --- | --- | --- | --- | --- | --- | --- | --- | --- | --- | --- | --- | --- |
|  | **NRRL Y-11430**  **+RPP0 +KAR2 +PDI +HAC1** | **NRRL Y-11430**  **(MeOH-free)** | **NRRL Y-11430**  **+HAC1**  **(MeOH-free)** | **NRRL Y-11430**  **+RPP0 +KAR2 +PDI +HAC1** | **NRRL Y-11430**  **(MeOH-free)** | **NRRL Y-11430**  **+HAC1**  **(MeOH-free)** | **NRRL Y-11430**  **+RPP0 +KAR2 +PDI +HAC1** | **NRRL Y-11430**  **(MeOH-free)** | **NRRL Y-11430**  **+HAC1**  **(MeOH-free)** | **NRRL Y-11430**  **+RPP0 +KAR2 +PDI +HAC1** | **NRRL Y-11430**  **(MeOH-free)** | **NRRL Y-11430**  **+HAC1**  **(MeOH-free)** | **NRRL Y-11430**  **+RPP0 +KAR2 +PDI +HAC1** | **NRRL Y-11430**  **(MeOH-free)** | **NRRLY-11430**  **+HAC1**  **(MeOH-free)** | **M kDa** |
|  |  |  |  |  |  |  |  |  |  |  |  |  |  |  | 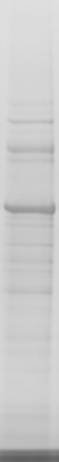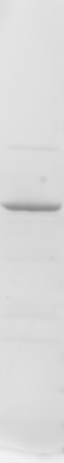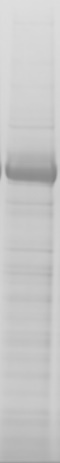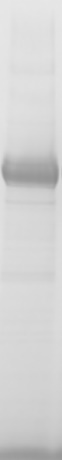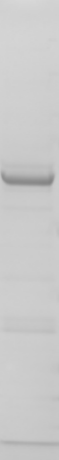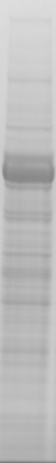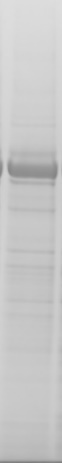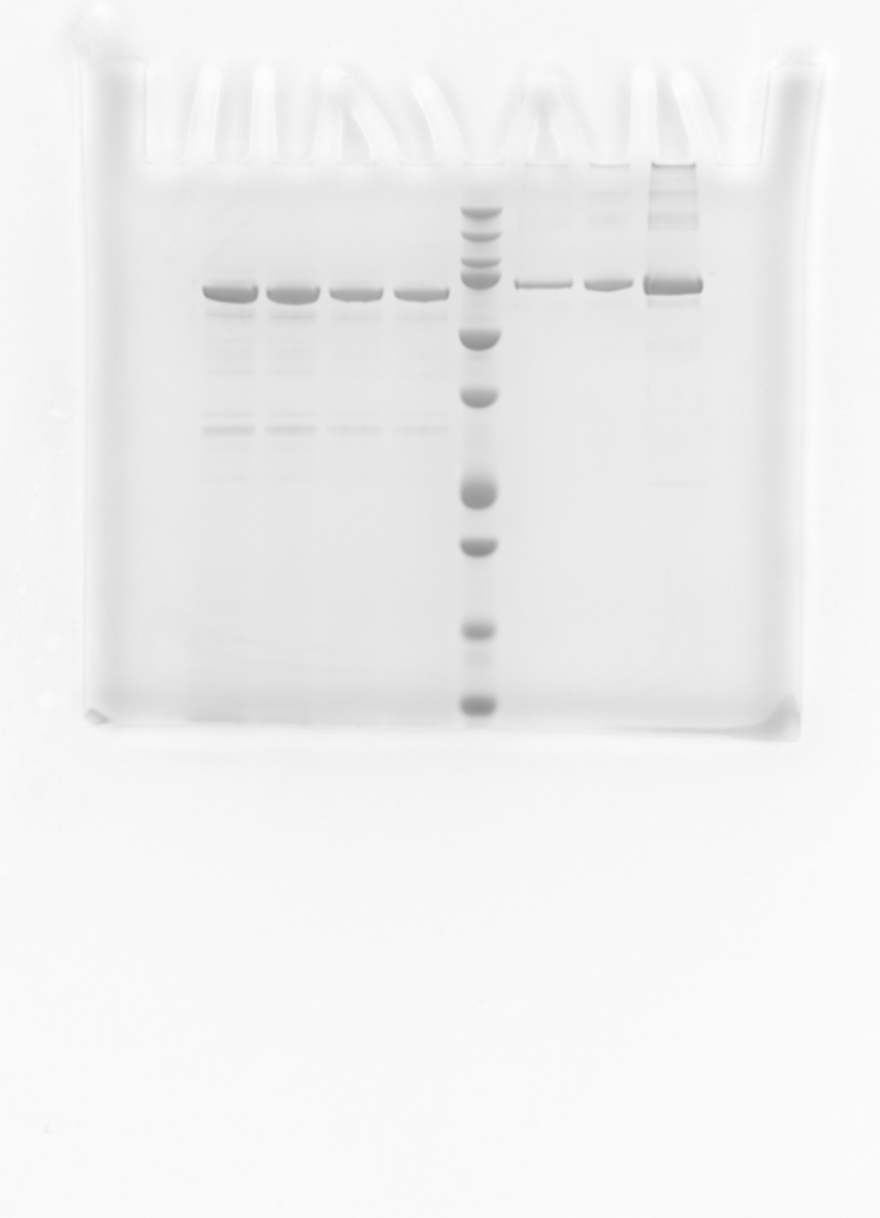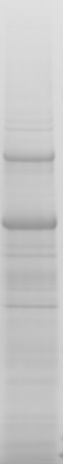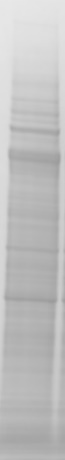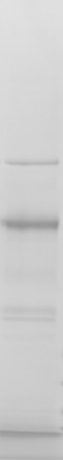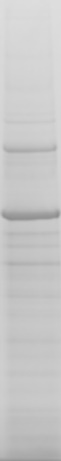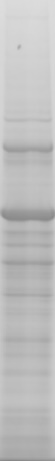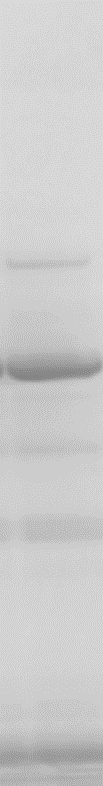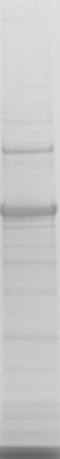  *****  *****  *****  *****  *****  *****  *****  *****  *****  *****  *****  *****  *****  *****  ***** | 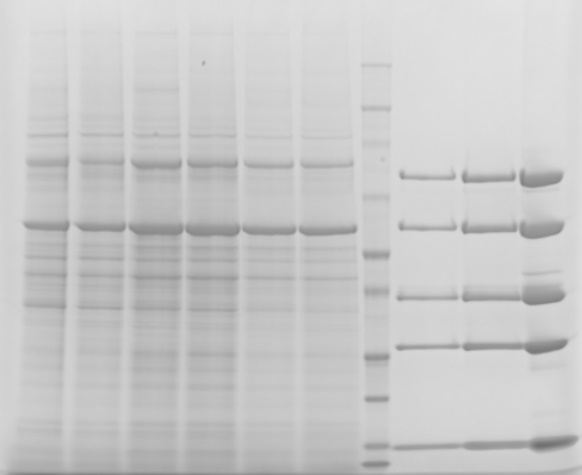  - 15  - 160  - 110  - 80  - 60  - 50  - 40  - 30  - 20 |
| **Titer (g.l^-1^)** | **2.2** | **0.8** | **1.6** | **3.1** | **0.5** | **1.0** | **3.0** | **1.5** | **2.6** | **3.3** | **1.6** | **4.0** | **3.5** | **1.5** | **2.9** |  |
